# Folic Acid Combined with Melatonin Might Prevent Hepatic Steatosis by Alleviating Endoplasmic Reticulum Stress to Promote Lipid Droplet Lipolysis in High-Fat Diet-Fed Mice

**DOI:** 10.3390/nu17233641

**Published:** 2025-11-21

**Authors:** Dan Sun, Yanzhen Ma, Yixian Bai, Xue Bai, Weiheng Liu, Lin Du, Peng Wang, Xi Liang, Hui Liang, Huaqi Zhang

**Affiliations:** Department of Nutrition and Food Hygiene, School of Public Health, Qingdao University, 308 Ningxia Road, Qingdao 266071, China; sundan202409@163.com (D.S.); mayanzhen97@163.com (Y.M.); bai_yixian@163.com (Y.B.); snowxue216@126.com (X.B.); 18648646729@163.com (W.L.); dl15163129157@163.com (L.D.); wpeng@qdu.edu.cn (P.W.); liangxi6029@163.com (X.L.); qdlianghui@126.com (H.L.)

**Keywords:** folic acid, melatonin, lipid droplets, lipolysis, endoplasmic reticulum stress, hepatic steatosis

## Abstract

**Objectives**: The purpose of this study is to observe the preventive effect of folic acid (FA) combined with melatonin (MLT) on high-fat diet (HFD)-induced hepatic steatosis and to explore its potential mechanism. **Methods**: Fifty male C57BL/6J mice were randomized into five groups: control group (CN), high-fat diet group (HD), FA supplementation group (HF), MLT supplementation group (HM), and FA combined with MLT supplementation group (HFM). The experiment lasted for 10 weeks. **Results**: FA combined with MLT effectively inhibited HFD-induced increases in liver index, hepatic TG levels, and serum TG levels. Histological examination revealed that FA combined with MLT significantly reduced the area of hepatic steatosis and the accumulation of lipid droplets (LD). Western blot analysis showed FA combined with MLT activated AMPK, inhibiting the expression of ERS-related proteins, thereby reducing the expression of LD lipolysis-associated proteins. **Conclusions**: FA combined with MLT might prevent HFD-induced hepatic steatosis by attenuating ERS and subsequently promoting LD lipolysis.

## 1. Introduction

Metabolic dysfunction-associated steatotic liver disease (MASLD), formerly known as non-alcoholic fatty liver disease (NAFLD) [1,2], has emerged as one of the major causes of chronic liver disease worldwide. Recent epidemiological studies demonstrate a global adult prevalence of 38% for MASLD, whereas the Asian prevalence is estimated at 36.91% [3,4]. Projections indicate that the global MASLD prevalence may escalate to 55.2% by 2040 [5]. Hepatic steatosis, recognized as the hallmark feature and initial pathological stage of MASLD [6], may serve as a critical link for controlling the progression of MASLD.

Excessive accumulation of lipid droplets (LD) in hepatocytes is a hallmark of hepatic steatosis, and promoting LD lipolysis is one of the effective approaches to preventing the occurrence of hepatic steatosis. As highly dynamic organelles, LD are surrounded by a phospholipid monolayer, which contains perilipin (Plin) family [7,8,9]. Perilipin 2 (Plin2/ADRP) and perilipin 5 (Plin5/MLDP), the two key members of the Plin family, have been demonstrated to participate in LD lipolysis. Plin2 is a good general marker for LD accumulation, which can form a physical barrier on the surface of LD, blocking adipose triglyceride lipase (ATGL) contact with LD and thereby inhibiting LD lipolysis [10,11]. Plin5 is a crucial factor promoting the contact between LD and mitochondria [12]. It binds to both ATGL and its coactivator comparative gene identification 58 (CGI-58), thereby attenuating ATGL activity and inhibiting LD lipolysis [13]. Therefore, overexpression levels of Plin2 and Plin5 can interfere with the function of ATGL and CGI-58, thereby inhibiting LD lipolysis. In contrast, inhibiting the excessive expression of Plin2 and Plin5 can promote LD lipolysis, thereby reducing the hepatic LD accumulation.

Endoplasmic reticulum stress (ERS) can regulate peroxisome proliferator-activated receptor gamma (PPARγ) expression and subsequently impact Plin2 and Plin5 expression. A high-fat diet (HFD), hypoxia, and other stimuli can disrupt ER homeostasis and lead to the accumulation of unfolded and misfolded proteins in the ER lumen, consequently inducing ERS [14]. In response to this adverse condition, the protein kinase RNA-like ER kinase (PERK) is activated and then triggers the unfolded protein response (UPR) by phosphorylating its downstream factors eukaryotic translation initiation factor 2α (eIF2α), activating transcription factor 4 (ATF4), and activating the CCAAT/enhancer-binding protein homologous protein (CHOP) [15]. Among these, CHOP, as a downstream factor of this signaling pathway, has been well confirmed to further regulate PPARγ expression [16]. Plin2, as a target gene of PPARγ, exhibited significantly upregulated expression in arachidonic acid-treated SZ95 cells, and this upregulation was completely abolished by the administration of a PPARγ-specific antagonist [17]. Similarly, inhibition of PPARγ activity also prevents the promotive effects of curcumin on Plin5 expression and LD formation in hepatic stellate cells [18]. Therefore, ameliorating ERS to enhance PPARγ and promote lipolysis of LD mediated by Plin2 and Plin5 may be an effective way to prevent hepatic steatosis.

Folic acid (FA) has been reported to possess physiological functions such as antioxidants, regulate inflammation, and improve hyperhomocysteinemia and related endothelial dysfunction [19,20,21]. Melatonin (MLT) is widely recognized for its role in regulating circadian rhythms and alleviating insomnia and jet lag. MLT also demonstrates antioxidant, anti-inflammatory, and immunomodulatory properties [22,23]. Given that both FA and MLT have significant antioxidant properties, their combined administration has been investigated in several animal studies. It was found that the combination of FA and MLT was more effective in inhibiting oxidative stress than their individual administrations, and symptoms of diabetic nephropathy and carbon tetrachloride (CCl_4_)-induced hepatic injury were also alleviated more significantly [24,25]. An emerging study has demonstrated that FA combined with MLT exhibits superior efficacy than either alone in alleviating reserpine-induced fibromyalgia by reducing mast cell infiltration and microglial activation, thereby attenuating pain hypersensitivity and depressive-like behaviors [26]. In addition to their antioxidant and anti-inflammatory effects, both FA and MLT have also been found to regulate lipid metabolism. Recent clinical evidence confirms that FA supplementation significantly reduces serum triglyceride (TG) levels in patients with metabolic diseases, demonstrating a notable lipid-improving effect [27]. Furthermore, animal and human studies support the role of MLT in lipid metabolism and highlight its potential in the treatment of hepatic steatosis [28,29]. However, whether the combination of FA and MLT exerts superior efficacy in ameliorating hepatic steatosis remains to be explored.

This study is the first to evaluate the preventive effect of 5 mg/kg FA combined with 10 mg/kg MLT on hepatic steatosis in HFD-fed mice, while also exploring its underlying mechanism through ERS-mediated LD lipolysis.

## 2. Materials and Methods

### 2.1. Reagents and Chemicals

MLT was procured from Sigma Aldrich Chemical Co. (CAS-NO:73-31-4, St. Louis, MO, USA). The rodent diet was procured from Wuxi Fanbo Biotechnology Co., Ltd. (Wuxi, China). Primary antibodies for Western blotting in this study included AMPK, p-AMPK, ATGL, and PPARγ were procured from Cell Signaling Technology (Danvers, MA, USA). GRP78, PERK, p-PERK, eIF2α, p-eIF2α, ATF4, CGI-58, and CHOP were procured from Abcam (Cambridge, UK). Plin2, Plin5, and β-actin were procured from ABclonal Biotechnology (Wuhan, China). Corresponding secondary antibodies were procured from Amersham (Bucks, UK).

### 2.2. Animals

A total of 50 six-week-old male C57BL/6J mice (20 ± 2 g) were procured from Beijing Huafukang Biotechnology Co. (Beijing, China). All mice were raised in the Biomedical Center of Qingdao University, where they were maintained under a controlled environment (20–25 °C, 40–50% humidity, 12-h light/dark cycle) and provided free access to food and water. All animal experiments were conducted in accordance with the ARRIVE guidelines and with the approval of the Qingdao University Laboratory Animal Welfare Ethics Committee (approval number: 20231102C577520240113053, approval date 15 October 2023). Sample size was determined to ensure adequate power while minimizing animal usage. Every effort was made to minimize suffering.

### 2.3. Experimental Design

**Figure 1:**
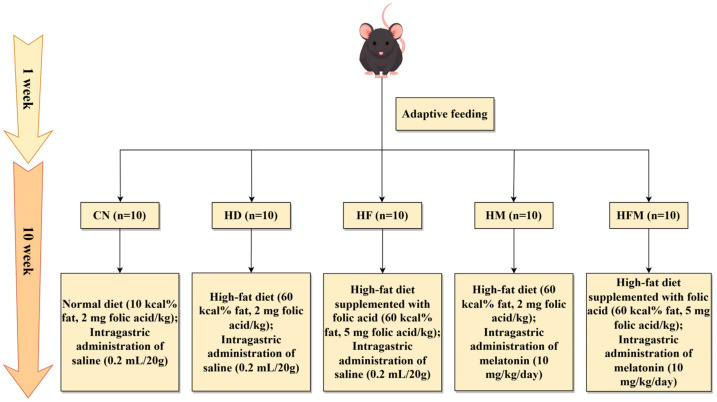
Experimental grouping and interventions of the study.

Following 1-week acclimatization, the mice were randomly assigned to five groups (*n* = 10 per group): control group (CN), high-fat diet group (HD), FA supplementation group (HF), MLT supplementation group (HM), and FA combined with MLT supplementation group (HFM). All groups except the CN were fed a HFD. The CN, HD, and HF were administered saline (0.2 mL/20 g/day) by oral gavage. The HM and HFM were administered MLT (10 mg/kg/day dissolved in saline) via oral gavage. FA was supplemented in the diet at a dose of 2 mg/kg for the CN, HD, and HM, and at 5 mg/kg for the HF and HFM. The specific intervention protocol is illustrated in Figure 1, and the detailed dietary formulations are provided in Table S1.

The body weight of the mice in each group was monitored weekly for 10 weeks. After 12 h of fasting, mice were anesthetized [30]. Mice were euthanized after blood collection, and this was followed by immediate excision of the liver, inguinal white adipose tissue (iWAT), and epididymal white adipose tissue (eWAT). The blood samples were centrifuged at 3000× *g* for 10 min at 4 °C to obtain the serum. The liver samples were weighed for accurate calculation of the liver index (liver weight (g)/body weight (g) × 100%). Then, partial liver and adipose tissue (iWAT and eWAT) samples were fixed in 4% paraformaldehyde for hematoxylin and eosin (H&E) staining and immunohistochemistry (IHC). Another portion of the liver tissue samples was cryo-embedded in Tissue-Tek OCT compound (Sakura Finetek, Torrance, CA, USA) for subsequent Oil Red O (ORO) staining. The serum and remaining liver, iWAT, and eWAT samples were stored at −80 °C for further experiments.

### 2.4. Serum Biochemical Analysis

Serum levels of alanine aminotransferase (ALT), aspartate aminotransferase (AST), TG, total cholesterol (TC), high-density lipoprotein-cholesterol (HDL-C), and low-density lipoprotein-cholesterol (LDL-C) were detected using an automated biochemical analyzer (Beckman, Los Angeles, CA, USA).

### 2.5. Hepatic Biochemical Analysis

Liver tissue samples were homogenized in pre-cooled saline (1:9, *w*/*v*) and subsequently subjected to centrifugation at 2500× *g* for 15 min (4 °C). The supernatants were collected and subsequently analyzed for TG, non-esterified fatty acid (NFFA), TC, malondialdehyde (MDA), superoxide dismutase (SOD), glutathione peroxidase (GSH-Px), and catalase (CAT) levels using commercial kits from Jiancheng Bioengineering Institute (Nanjing, China).

### 2.6. Histology Analysis

For H&E staining, liver and adipose tissue (iWAT and eWAT) samples fixed in 4% paraformaldehyde were embedded in paraffin blocks. Then, 4 μm serial sections were prepared, deparaffinized in xylene, rehydrated in graded ethanol, and stained with H&E (Solarbio, Beijing, China). Histopathological alterations were detected under an Olympus microscope (BX60, Olympus, Tokyo, Japan). Additionally, to evaluate hepatic lipid accumulation, fresh liver tissues were frozen in Tissue-Tek OCT compound and sectioned (10 μm) on a cryostat. Then cryosections were stained with ORO (Solarbio, Beijing, China), and images were acquired using an Olympus microscope (BX60, Olympus, Tokyo, Japan). Quantitative analysis of LD accumulation was performed using ImageJ software (v1.8.0, NIH, Bethesda, MD, USA), including both the steatosis area and the ORO staining positive area within the hepatic tissue.

### 2.7. Immunohistochemistry

The experimental protocols were conducted according to our previous methodology [31]. In brief, paraffin-embedded liver sections were routinely deparaffinized, rehydrated, and then incubated with 3% H_2_O_2_ at room temperature for endogenous peroxidase blockade. The sections were then incubated overnight at 4 °C with primary antibodies against Plin2 and Plin5 (1:200 dilution). After that, sections were washed thoroughly with Tris-buffered saline and then incubated with corresponding secondary antibodies at 37 °C for 30 min. Finally, sections were incubated with diaminobenzidine for color development and counterstained with hematoxylin. Digital images of the stained sections were captured with an Olympus BX60 microscope (Japan), and quantitative morphometric analysis was conducted using ImageJ software (v1.8.0, NIH, Bethesda, MD, USA).

### 2.8. Western Blot

Western blot analysis was done following our standardized protocol as described in previous studies [32,33]. The hepatic protein expression of AMPK, p-AMPK, GRP78, PERK, p-PERK, eIF-2α, p-eIF-2α, ATF4, CHOP, PPARγ, Plin2, Plin5, ATGL, and CGI-58 was determined by Western blot analysis. β-Actin served as an internal control.

### 2.9. Statistical Analysis

Data are presented as mean ± standard deviation (SD). Statistical analysis was conducted with SPSS 27.0 (SPSS, Chicago, IL, USA). Inter-group differences were assessed by ANOVA, and Fisher’s LSD test was used for post hoc comparisons when significant differences were found. *p* < 0.05 was considered statistically significant. The figures were prepared using GraphPad Prism 10 and Figdraw 2.0.

## 3. Results

### 3.1. Effects of FA Combined with MLT on Body Weight and Food Intake

**Figure 2:**
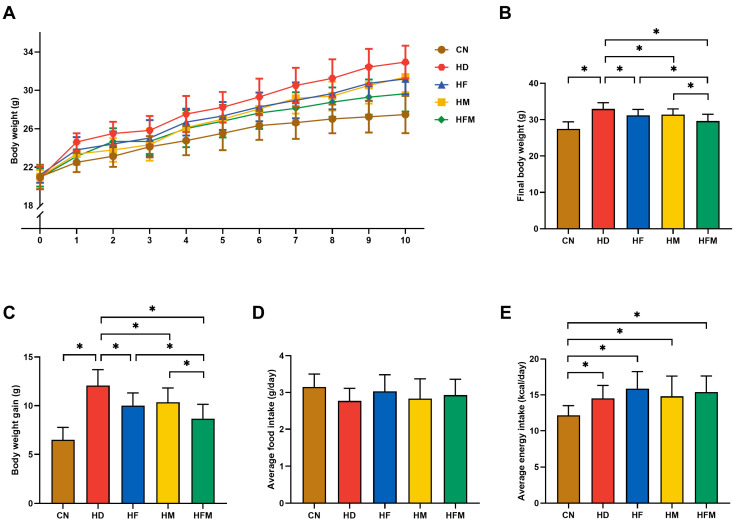
Effects of FA and MLT on body weight and food intake in mice. (**A**) Body weight, (**B**) Final body weight, (**C**) Body weight gain, (**D**) Average daily food intake, (**E**) Average daily energy intake. Values are presented as mean ± SD (*n* = 10). * indicates a significant difference at *p* < 0.05.

No significant difference in initial body weight was detected among the groups (*p* > 0.05; Figure 2A). As demonstrated in Figure 2B,C, final body weight and body weight gain were significantly elevated in the HD relative to the CN during the experiment. Significant reductions in both final body weight and weight gain were detected in the HF, HM, and HFM relative to the HD, with the most pronounced reduction detected in the HFM (*p* < 0.05). No significant difference was detected between the HF and HM (*p* > 0.05). Despite comparable daily average food intake across all five groups, the four HFD groups exhibited significantly higher daily average energy intake than the CN, with no significant difference detected among them (*p* > 0.05; Figure 2D,E).

### 3.2. Effects of FA Combined with MLT on Liver Index and Adipose Index

**Figure 3:**
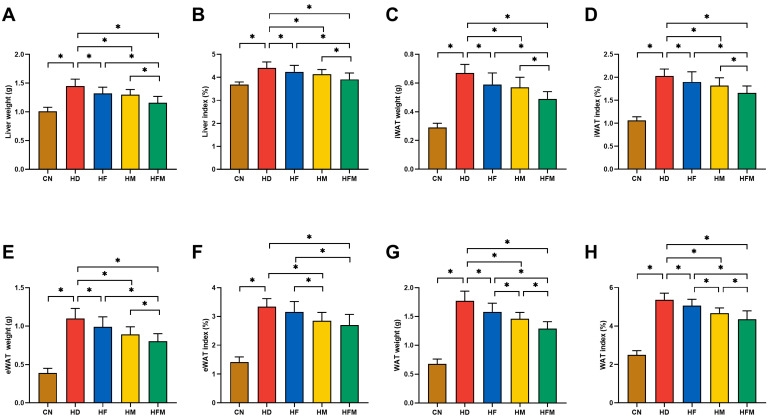
Effects of FA and MLT on liver index and adipose index. (**A**) Liver weight, (**B**) Liver index, (**C**) iWAT weight, (**D**) iWAT index, (**E**) eWAT weight, (**F**) eWAT index, (**G**) WAT weight, (**H**) WAT index. Values are presented as mean ± SD (*n* = 10). * indicates a significant difference at *p* < 0.05.

As demonstrated in Figure 3A,B, the HD exhibited a significant increase in liver weight and liver index relative to the CN. Significant reductions in liver weight and liver index were detected in the HF, HM, and HFM relative to the HD, with the most pronounced reduction detected in the HFM (*p* < 0.05). No significant difference was detected between the HF and HM (*p* > 0.05).

Significant increases in iWAT weight, iWAT index, eWAT weight, eWAT index, WAT weight, and WAT index were detected in the HD relative to the CN. Significant reductions in the above parameters were detected in the HF, HM, and HFM relative to the HD, with the most pronounced reduction detected in the HFM (*p* < 0.05; Figure 3C–H). No significant difference was detected between the HF and HM (*p* > 0.05; Figure 3C–H).

### 3.3. Effects of FA Combined with MLT on Serological Indicators

**Figure 4:**
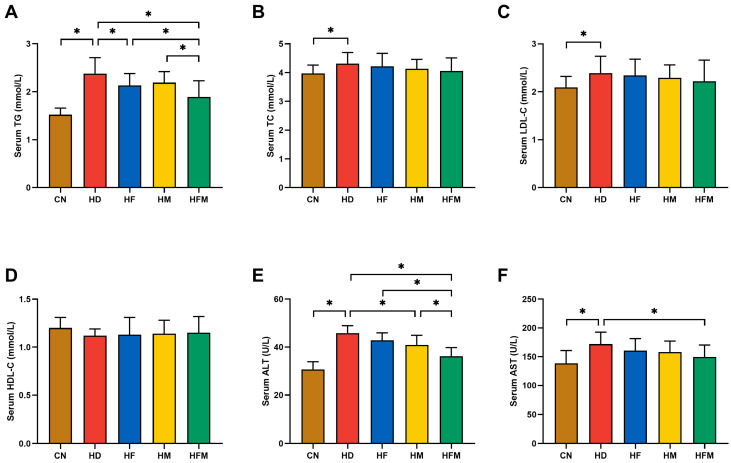
Effects of FA and MLT on serological indicators. (**A**) Serum TG, (**B**) Serum TC, (**C**) Serum LDL-C, (**D**) Serum HDL-C, (**E**) Serum ALT, (**F**) Serum AST. Values are presented as mean ± SD (*n* = 10). * indicates a significant difference at *p* < 0.05.

Serum TG was significantly elevated in the HD than in the CN. A significant reduction in serum TG was detected in the HF, HM, and HFM relative to the HD, with the most pronounced reduction detected in the HFM (*p* < 0.05; Figure 4A). No significant difference in serum TG was detected between the HF and HM (*p* > 0.05; Figure 4A). As demonstrated in Figure 4B,C, the HD exhibited significantly higher serum TC and LDL-C relative to the CN (*p* < 0.05). No significant difference in serum HDL-C was detected among the five groups (*p* > 0.05; Figure 4D). The HD demonstrated significantly elevated serum ALT and AST relative to the CN. Compared with the HD, HF, HM, and HFM showed reduced serum ALT and AST, with the most pronounced reduction detected in the HFM (*p* < 0.05; Figure 4E,F). No significant difference in serum ALT and AST was detected between the HF and HM (*p* > 0.05; Figure 4E,F).

### 3.4. Effects of FA Combined with MLT on Pathological Changes of Hepatic and Adipose Tissues

**Figure 5:**
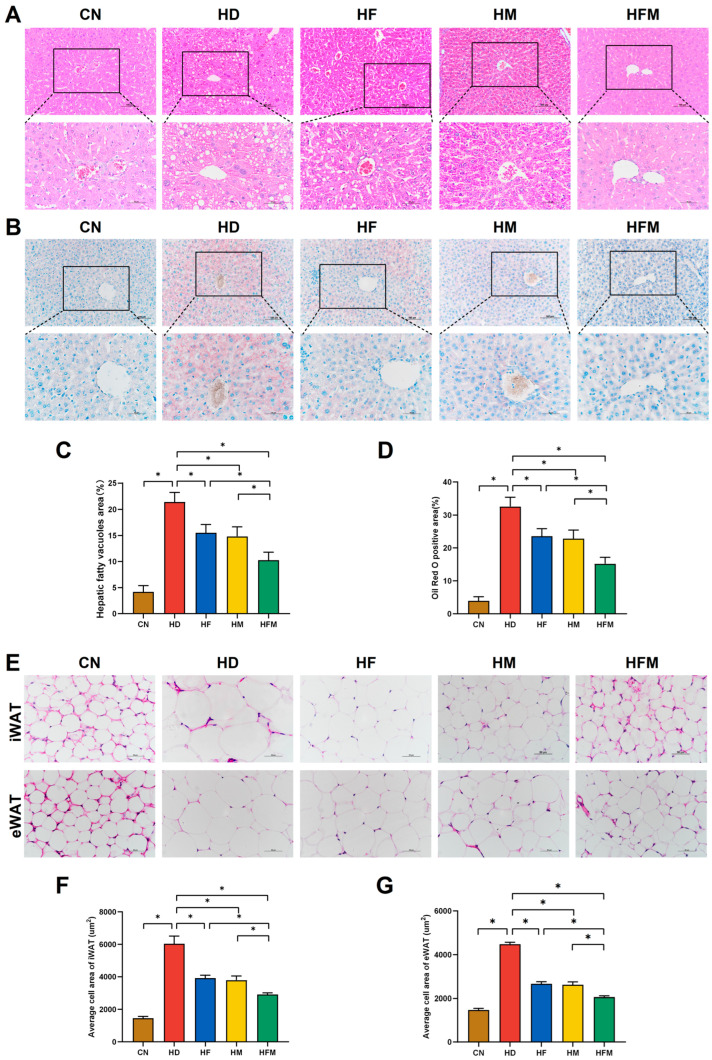
Effects of FA combined with MLT on pathological changes of hepatic and adipose tissues. (**A**) Representative H&E staining images of liver tissues (20×, scale bars = 100 µm; 40×, scale bars = 50 µm), (**B**) Representative ORO staining images of liver tissues (20×, scale bars = 100 µm; 40×, scale bars = 50 µm), (**C**) Percentage of hepatic steatosis area (*n* = 10), (**D**) Percentage of ORO positive area (*n* = 10), (**E**) H&E staining of iWAT tissues and eWAT tissues (20×, scale bars = 100 µm), (**F**) Average cell area of iWAT (*n* = 10), (**G**) Average cell area of eWAT (*n* = 10). Values are presented as mean ± SD. * indicates a significant difference at *p* < 0.05.

The results of liver H&E staining are presented in Figure 5A. A significant increase in the hepatic steatosis area was detected in the HD relative to the CN. Significant reductions in the hepatic steatosis area were detected in the HF, HM, and HFM relative to the HD, with the most pronounced reduction detected in the HFM (*p* < 0.05; Figure 5C). No significant difference in the hepatic steatosis area was detected between the HF and HM (*p* > 0.05; Figure 5C). The results of liver ORO staining are presented in Figure 5B. A significant increase in the hepatic ORO positive area was detected in the HD relative to the CN. Significant reductions in the hepatic ORO positive area were detected in the HF, HM, and HFM relative to the HD, with the most pronounced reduction detected in the HFM (*p* < 0.05; Figure 5D). No significant difference in the hepatic ORO positive area was detected between the HF and HM (*p* > 0.05; Figure 5D).

H&E staining of white adipose tissues are shown in Figure 5E. The average cell areas of iWAT and eWAT were significantly increased in the HD relative to the CN. Compared with the HD, the average cell areas of iWAT and eWAT were significantly reduced in the HF, HM, and HFM, with the most pronounced reduction detected in the HFM (*p* < 0.05; Figure 5F,G). No significant difference in the average cell areas of iWAT and eWAT was detected between the HF and HM (*p* > 0.05; Figure 5F,G).

### 3.5. Effects of FA Combined with MLT on Hepatic Lipid Levels and Oxidative Stress Levels

**Figure 6:**
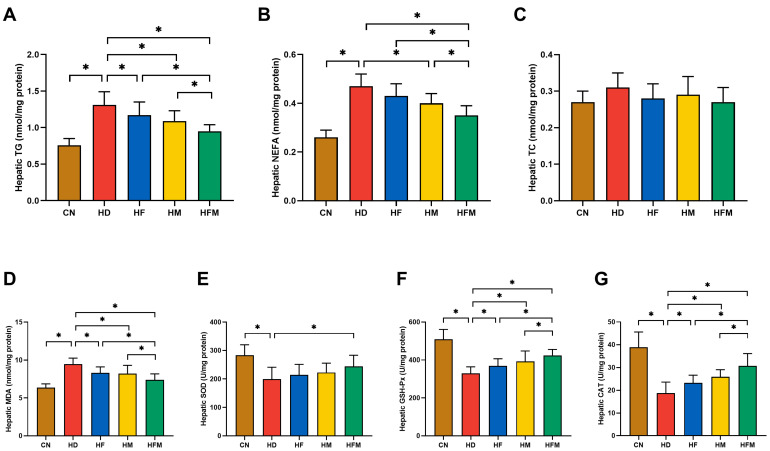
Effects of FA combined with MLT on hepatic lipid levels and oxidative stress levels. (**A**) Hepatic TG, (**B**) Hepatic NEFA, (**C**) Hepatic TC, (**D**) Hepatic MDA, (**E**) Hepatic SOD, (**F**) Hepatic GSH-Px, (**G**) Hepatic CAT. Values are presented as mean ± SD (*n* = 10). * indicates a significant difference at *p* < 0.05.

As demonstrated in Figure 6A,B, hepatic TG and NEFA levels were significantly increased in the HD relative to the CN. Significant reductions in hepatic TG and NEFA levels were detected in the HF, HM, and HFM relative to the HD, with the most pronounced reduction detected in the HFM (*p* < 0.05). No significant difference in hepatic TG and NEFA levels was detected between the HF and HM (*p* > 0.05). Regarding hepatic TC level, no significant difference was detected among the five groups (*p* > 0.05; Figure 6C).

As demonstrated in Figure 6D, hepatic MDA level was significantly elevated in the HD relative to the CN. Significant reduction in hepatic MDA level was detected in the HF, HM, and HFM relative to the HD, with the most pronounced reduction detected in the HFM (*p* < 0.05). No significant difference in hepatic MDA level was detected between the HF and HM (*p* > 0.05). Furthermore, as illustrated in Figure 6E–G, compared with the CN, the HD exhibited significantly lower activities of hepatic SOD, GSH-Px, and CAT. Compared with the HD, the activities of the above antioxidant enzymes were significantly elevated in the HF, HM, and HFM, with the most pronounced elevation detected in the HFM (*p* < 0.05). No significant difference in the activities of hepatic SOD, GSH-Px, and CAT was detected between the HF and HM (*p* > 0.05).

### 3.6. Effects of FA Combined with MLT on the Expression of Proteins Related to ERS

**Figure 7:**
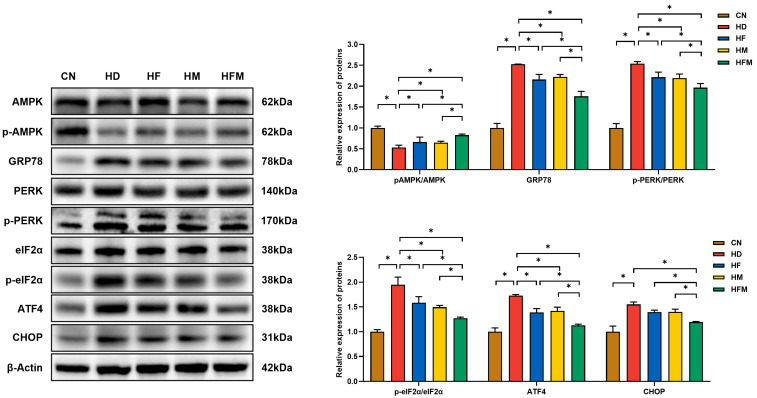
Effects of FA combined with MLT on the expression of proteins related to hepatic ERS. Values are presented as mean ± SD (*n* = 3). * indicates a significant difference at *p* < 0.05.

As demonstrated in Figure 7, the expression of p-AMPK/AMPK was significantly reduced in the HD relative to the CN. A significant elevation in p-AMPK/AMPK expression was detected in the HF, HM, and HFM relative to the HD, with the most pronounced elevation detected in the HFM (*p* < 0.05). No significant difference in p-AMPK/AMPK expression was detected between the HF and HM (*p* > 0.05). Furthermore, the expression levels of GRP78, p-PERK/PERK, p-eIF2α/eIF2α, ATF4, and CHOP were significantly increased in the HD relative to the CN. The levels of these proteins were significantly reduced in the HF, HM, and HFM relative to the HD, with the most pronounced reduction detected in the HFM (*p* < 0.05). No significant difference in the levels of these proteins was detected between the HF and HM (*p* > 0.05).

### 3.7. Effects of FA Combined with MLT on the Expression of Proteins Related to Hepatic LD Lipolysis

**Figure 8:**
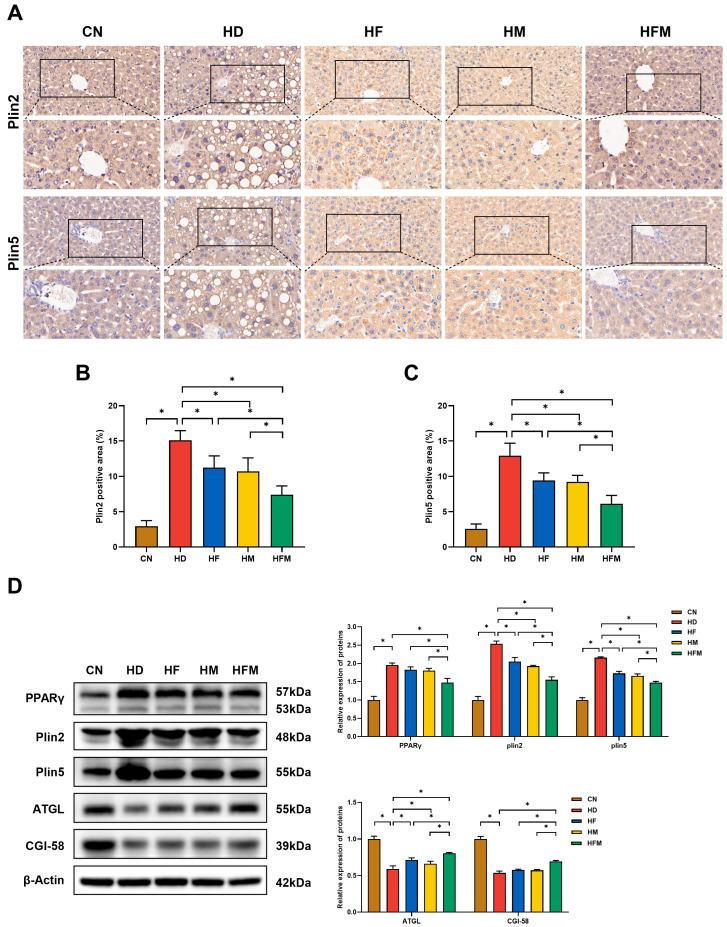
Effects of FA combined with MLT on the expression of proteins related to hepatic LD lipolysis. (**A**) Representative hepatic IHC images of Plin2 and Plin5 (40×, scale bars = 20 µm; 63×, scale bars = 20 µm), (**B**) The positive area of Plin2 (*n* = 10), (**C**) The positive area of Plin5 (*n* = 10), (**D**) The expression levels of hepatic LD lipolysis related proteins (*n* = 3). Values are presented as mean ± SD. * indicates a significant difference at *p* < 0.05.

The IHC results of hepatic Plin2 and Plin5 are shown in Figure 8A. The expression levels of Plin2 and Plin5 were significantly elevated in the HD relative to the CN. Significant reductions in the levels of Plin2 and Plin5 were detected in the HF, HM, and HFM relative to the HD, with the most pronounced reduction in the HFM (*p* < 0.05; Figure 8B,C). No significant difference in the levels of Plin2 and Plin5 was detected between the HF and HM (*p* > 0.05; Figure 8B,C). Consistent with these results, Western blot analysis further confirmed the changes in the expression levels of Plin2 and Plin5. As demonstrated in Figure 8D, Western blot results indicated that the change in PPARγ expression was similar with Plin2 and Plin5. Additionally, compared with the CN, the expression levels of ATGL and CGI-58 were significantly reduced in the HD. The levels of ATGL and CGI-58 were significantly increased in the HF, HM, and HFM relative to the HD, with the most pronounced increase detected in the HFM (*p* < 0.05). No significant difference in the levels of ATGL and CGI-58 was detected between the HF and HM (*p* > 0.05).

## 4. Discussion

**Figure 9:**
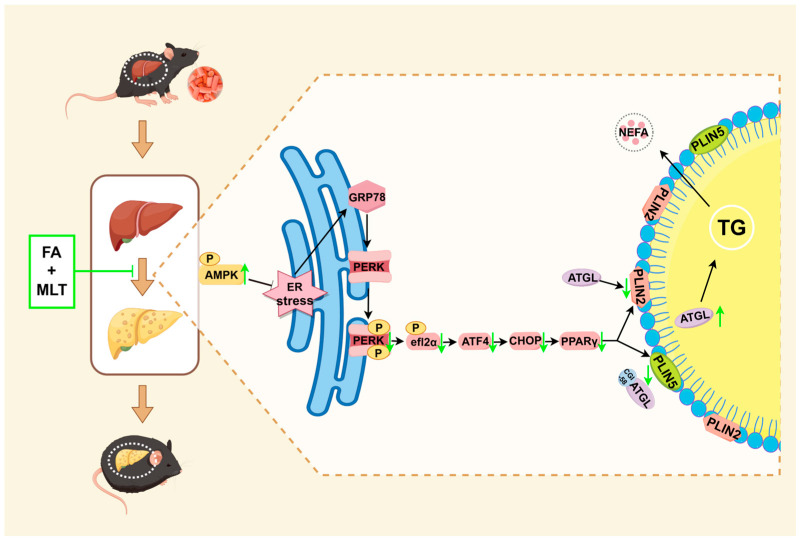
Potential mechanisms of FA combined with MLT in preventing hepatic steatosis.

This study provides the first demonstration that combined administration of FA and MLT exhibits superior efficacy in preventing HFD-induced hepatic steatosis. Mechanistically, the combination of FA and MLT might alleviate hepatic ERS by activating AMPK, thereby enhancing PPARγ-mediated lipolysis of LD, ultimately reducing lipid accumulation and preventing hepatic steatosis (Figure 9).

Hepatic steatosis is a reversible pathological stage, and it also serves as a critical link in the prevention and control of MASLD. The presence of numerous LD in the liver by histopathological examination is not only a typical feature of hepatic steatosis, but also the gold standard for hepatic steatosis. Meanwhile, hepatic steatosis in both humans and animals may be accompanied by dyslipidemia [34,35,36]. HFD feeding is a common method to establish a hepatic steatosis model [37]. In this study, we fed C57BL/6 mice with 60% HFD for 10 weeks. Histopathological examination revealed significant steatosis and excessive LD accumulation in the liver. In addition, the HD showed significant increases in the liver index, hepatic TG, NEFA levels, and serum TG levels. Hepatic steatosis can cause liver function damage, so we also measured serum ALT and AST levels and found their levels significantly elevated. Collectively, these results indicate that HFD-fed mice exhibited hepatic steatosis.

In view of the antioxidant properties of FA and MLT, some studies have evaluated their combined administration and found that the combination exhibits better biological effects. For example, the combination of FA (2.5 mg/kg) and MLT (10 mg/kg) can better ameliorate CCl_4_-induced hepatic injury in rats through its antioxidant and anti-inflammatory properties [25]. The combination of FA (100 mg/kg) and MLT (10 mg/kg) also ameliorates diabetic nephropathy in rats via suppression of oxidative stress and attenuation of lipid peroxidation [24]. Findings from human and animal research indicate that both FA and MLT are involved in the modulation of lipid metabolism [27,38,39]. However, it remains unclear whether their combined administration can effectively prevent hepatic steatosis, which sparked our interest.

In previous animal experiments, it was found that administering 10 mg/kg FA through feed significantly improved lipid metabolism disorders in mice [40]. In our previous studies, we found that administering 5 mg/kg FA through feed can significantly improve hepatic steatosis in pregnant rats fed a HFD [41] and prevent hepatic steatosis in their offspring exposed to a HFD [42]. Intragastric administration of 10–20 mg/kg MLT was found to regulate body adipose deposition and lipid metabolism [43] and restore hepatic lipid metabolic homeostasis [44]. Since this study is the first to observe the preventive effect of FA and MLT in combination on hepatic steatosis, we selected the relatively low effective intervention doses of 5 mg/kg FA and 10 mg/kg MLT. After 10 weeks of intervention, we found that the activity of key hepatic antioxidant enzymes was significantly elevated in HFM relative to the HD, HF, and HM. This result indicated that the combination of FA and MLT indeed exerts a stronger antioxidant effect, which is consistent with existing studies [24,25]. More importantly, relative to the HD, the hepatic LD accumulation was significantly reduced and hepatic and serum lipid profiles were significantly improved in the HF, HM, and HFM. Compared with the HM and HF, these improvements were most significant in HFM. These findings demonstrate that the combination of FA and MLT effectively prevents HFD-induced hepatic steatosis and exhibits superior efficacy relative to monotherapy.

The liver serves as the center of lipid metabolism. Long-term HFD exposure can disrupt hepatocyte homeostasis and trigger ERS. In response to this adverse condition, the UPR can be triggered by signaling branches from the ER transmembrane sensors PERK, IRE1, and ATF6 [45]. As one of the key branches of the UPR, the PERK-eIF2α-ATF4-CHOP pathway has been confirmed to serve a pivotal function in modulating hepatic lipid metabolism [46]. The GRP78 is a representative ERS marker [47]. Under basal conditions, PERK binds to GRP78 to maintain an inactive state. However, during ERS, GRP78 preferentially associates with unfolded proteins, and PERK dissociates from GRP78 and undergoes autophosphorylation, thereby phosphorylating eIF2α and activating ATF4. Under sustained ERS status, ATF4 further up-regulates the expression of CHOP [45,48]. CHOP can further modulate the expression of lipid metabolism-related proteins, thereby leading to lipid accumulation. Therefore, inhibiting the GRP78/PERK signaling pathway and subsequently reducing the expression of CHOP is crucial for improving liver lipid metabolism abnormalities.

AMPK has been extensively confirmed to alleviate ERS upon its activation in both in vivo and in vitro studies [49,50]. In human HepG2 cells and primary mouse hepatocytes, astragaloside IV alleviates free fatty acid (FFA)-induced ERS. However, upon pre-treatment of cells with the AMPK inhibitor Compound C prior to FFA exposure, it was found that the improvement effect of Astragaloside IV on ERS was significantly weakened [49]. Treatment with the AMPK activator A769662 significantly alleviated palmitic acid-induced ERS in mouse C2C12 myoblasts. Similarly, enhanced AMPK phosphorylation can reduce the expression of ERS markers in the type 2 diabetes mellitus animal model [50]. These results suggest that AMPK can negatively regulate lipid-induced ER stress in hepatocytes. Both FA and MLT have been well confirmed to activate AMPK in many previous in vivo and in vitro studies [51,52]. For example, palmitic acid can significantly suppresses AMPK phosphorylation in HepG2 cells, while FA and 5-MTHF treatment can effectively restore AMPK phosphorylation [51]. In bone marrow mesenchymal stem cells, H_2_O_2_ treatment significantly inhibited AMPK activity, while MLT pretreatment reversed this inhibitory effect and markedly increased AMPK phosphorylation levels [52]. Therefore, we speculate that the combination of FA with MLT may alleviate ERS and ultimately improve lipid metabolism disorders by regulating AMPK expression. This study revealed significantly suppressed hepatic AMPK phosphorylation in the HD relative to the CN, while the HF, HM, and HFM exhibited restored phosphorylation levels relative to the HD. Compared with HF and HM, HFM showed the best recovery effect on the hepatic AMPK phosphorylation level. Moreover, the hepatic expression of ERS-related proteins was significantly elevated in the HD relative to the CN. Compared with HD, the hepatic expression of these proteins was significantly decreased in the HF, HM, and HFM, with HFM showing the most significant decrease. The above findings suggest that the combination of FA with MLT may inhibit the GRP78/PERK signaling pathway by regulating the expression of AMPK, thereby alleviating hepatic ERS.

With the improvement of ERS, LD accumulation can also be alleviated. The downstream gene CHOP of the GRP78/PERK signaling pathway has been confirmed to regulate the expression of PPARγ [16]. Serving as a key regulator of lipid metabolism, PPARγ can regulate the expression of LD-associated proteins, thereby promoting LD lipolysis [53]. The excessive accumulation of LD is a typical feature of hepatic steatosis, and promoting LD lipolysis is one of the key ways to alleviate lipid accumulation. Various LD-associated proteins are embedded in the LD phospholipid monolayer, such as Plin family members Plin2 and Plin5 [54]. Plin2 serves as a well-established marker of LD accumulation and promotes fatty liver disease progression [10]. Plin5 promotes the contact of LD with mitochondria and actively participates in hepatic lipid metabolism [55]. Studies have demonstrated that both Plin2 and Plin5 are significantly upregulated in mice with HFD-induced hepatic steatosis and in patients with fatty liver [56,57,58,59]. In contrast, specific deficiency of either Plin2 or Plin5 reduces LD accumulation, ameliorates hepatic steatosis, and delays the progression of MASLD [60,61]. The expression of Plin2 and Plin5 was regulated by PPARγ in the liver [62,63]. In HFD-fed mice, swimming exercise negatively regulates PPARγ signaling and reduces Plin2 expression, thereby reducing hepatic fatty acid uptake and LD accumulation [62]. In vivo and in vitro studies have confirmed that sulforaphane reduces the expression of Plin2 and Plin5 by down-regulating PPARγ, thereby inhibiting LD maturation [63]. Furthermore, Plin2 and Plin5 have been confirmed to participate in LD lipolysis. Plin2 can cover the surface of LD and form a physical barrier that directly hinders the contact between ATGL and LD, thereby inhibiting LD lipolysis [11]. Plin5 can affect ATGL activity by competitive binding to ATGL and its coactivator CGI-58, then further prevent LD lipolysis [13]. This study revealed that the HD exhibited upregulated hepatic expression of PPARγ, Plin2, and Plin5 alongside downregulated expression of ATGL and CGI-58, relative to the CN. On the contrary, HF, HM, and HFM exhibited significantly lower expression of PPARγ, Plin2, and Plin5 concurrently with higher expression of ATGL and CGI-58, relative to the HD group. Compared with HF and HM, HFM demonstrated the lowest protein expression levels of PPARγ, Plin2, and Plin5, while exhibiting the highest levels of ATGL and CGI-58. In addition, we also detected the hepatic expression of Plin2 and Plin5 in mice by IHC. In line with Western blot findings, the combination of FA and MLT demonstrated the most pronounced downregulation of Plin2 and Plin5. These results suggest that FA combined with MLT can reduce hepatic lipid accumulation. The underlying mechanism may involve suppression of CHOP expression, which thereby attenuates the expression of PPARγ and its downstream targets Plin2 and Plin5, ultimately promoting LD lipolysis.

This study has several limitations. First, in order to mitigate the irritation caused by repeated gavage to experimental animals, FA was administered through a gentler approach of dietary supplementation. However, although dietary supplementation is one of the most common methods of FA intervention in animal studies, it cannot guarantee the consumption of an equal amount of FA by each mouse. Second, as a first-time exploration, only a single dose combination of FA and MLT was selected in this study. In future studies, the intervention dose can be adjusted to determine the optimal combination dose of FA and MLT for preventing hepatic steatosis. Currently, FA and MLT are used as dietary supplements to prevent fetal neural tube defects and improve sleep quality, respectively. Based on the safety of FA and MLT in population application, corresponding population intervention experiments can also be conducted in the future to further verify the preventive effect of FA combined with MLT on hepatic steatosis. It may provide a novel strategy for preventing hepatic steatosis.

## 5. Conclusions

This study observed that the combined administration of FA and MLT exerts a preventive effect on HFD-induced hepatic steatosis. It is the first to demonstrate that the combination of FA with MLT might alleviate ERS by activating AMPK, then down-regulating PPARγ and reducing the expression of Plin2 and Plin5, subsequently promoting LD lipolysis, decreasing LD accumulation, and ultimately achieving effective prevention of hepatic steatosis.

## Data Availability

The datasets used and/or analyzed in the current study can be obtained from the corresponding author upon reasonable request. Data availability is restricted in accordance with laboratory policies and confidentiality agreements.

## Supplementary Materials

The following supporting information can be downloaded at https://www.mdpi.com/article/10.3390/nu17233641/s1, Table S1: Composition and energy of the study diets. Figure S1: AMPK in the liver of Western blot. The molecular mass was 62 KD. Figure S2: p-AMPK in the liver of Western blot. The molecular mass was 62 KD. Figure S3: GRP78 in the liver of Western blot. The molecular mass was 78 KD. Figure S4: PERK in the liver of Western blot. The molecular mass was 140 KD. Figure S5: p-PERK in the liver of Western blot. The molecular mass was 170 KD. Figure S6: eIf-2α in the liver of Western blot. The molecular mass was 38 KD. Figure S7: p-eIf-2α in the liver of Western blot. The molecular mass was 38 KD. Figure S8: ATF4 in the liver of Western blot. The molecular mass was 38 KD. Figure S9: CHOP in the liver of Western blot. The molecular mass was 31 KD. Figure S10: β-actin in the liver of Western blot. The molecular mass was 42 KD. Figure S11: PPARγ in the liver of Western blot. The molecular mass were 57 KD and 53 KD. Figure S12: PLIN2 in the liver of Western blot. The molecular mass was 48 KD. Figure S13: PLIN5 in the liver of Western blot. The molecular mass was 55 KD. Figure S14: ATGL in the liver of Western blot. The molecular mass was 55 KD. Figure S15: CGI-58 in the liver of Western blot. The molecular mass was 39 KD. Figure S16: β-actin in the liver of Western blot. The molecular mass was 42 KD.
