# Supplementary material for: Folic Acid Combined with Melatonin Might Prevent Hepatic Steatosis by Alleviating Endoplasmic Reticulum Stress to Promote Lipid Droplet Lipolysis in High-Fat Diet-Fed Mice
Source: Nutrients. 2025 Nov 21;17(23):3641. doi: 10.3390/nu17233641 (PMC12693466; doi:10.3390/nu17233641)
Supplement: Supplementary file 1 [file nutrients-17-03641-s001.zip › nutrients-3975501-supplementary.pdf]

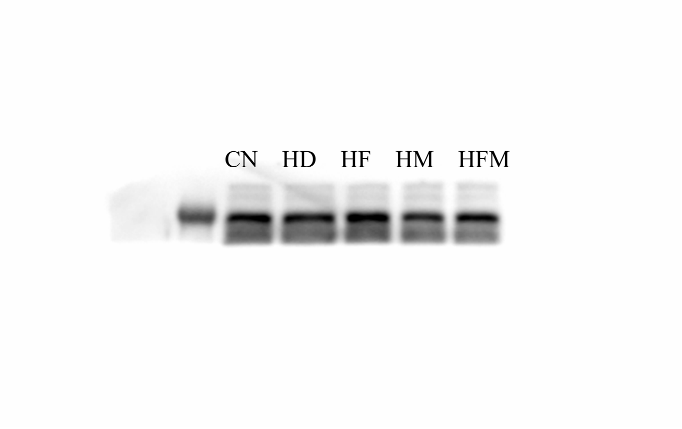

Figure S1. AMPK in the liver of Western blot. The molecular mass was 62 KD.

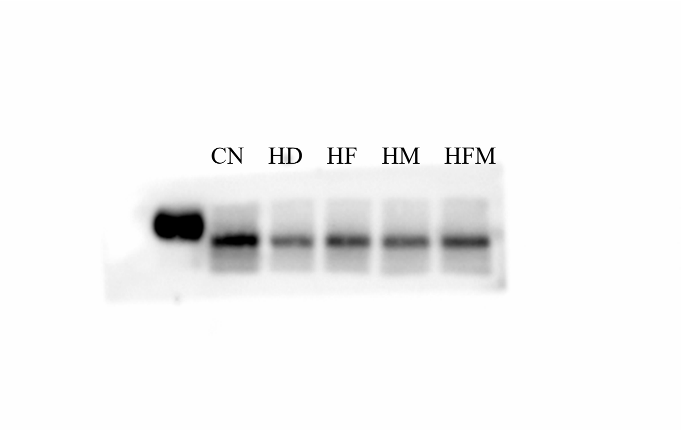

Figure S2. p-AMPK in the liver of Western blot. The molecular mass was 62 KD.

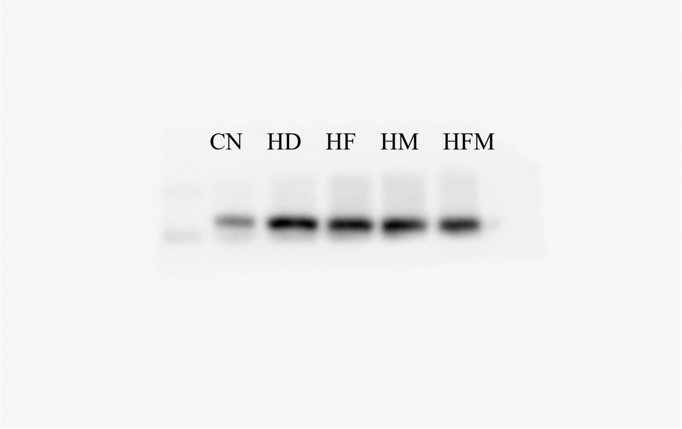

Figure S3. GRP78 in the liver of Western blot. The molecular mass was 78 KD.

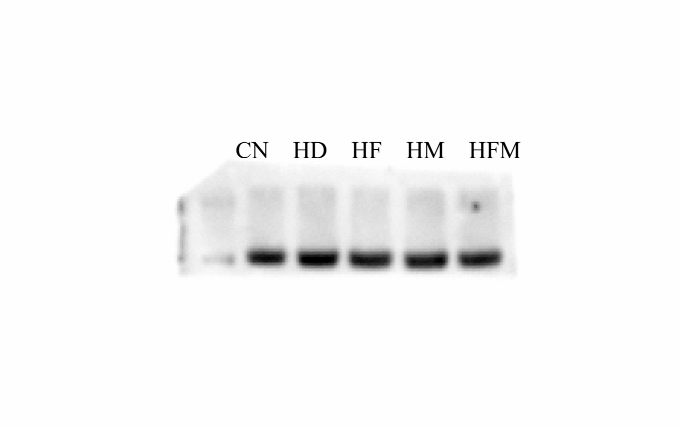

Figure S4. PERK in the liver of Western blot. The molecular mass was 140 KD.

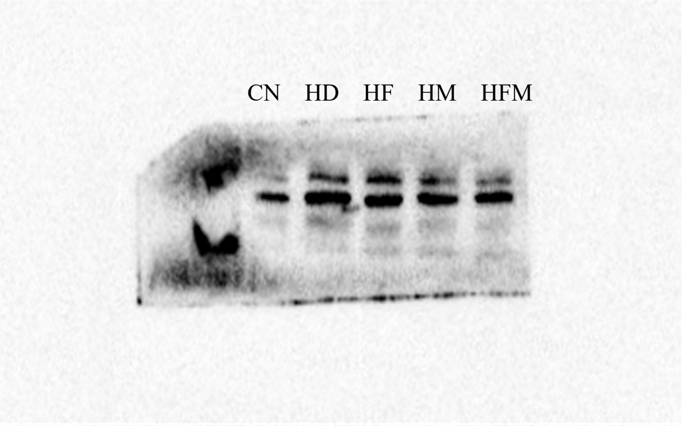

Figure S5. p-PERK in the liver of Western blot. The molecular mass was 170 KD.

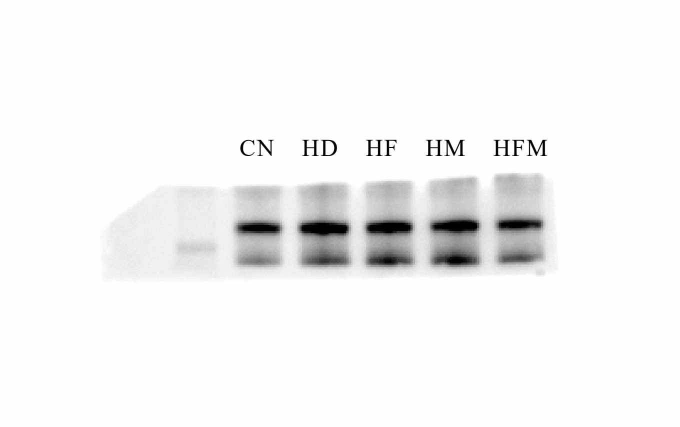

Figure S6. eIf-2α in the liver of Western blot. The molecular mass was 38 KD.

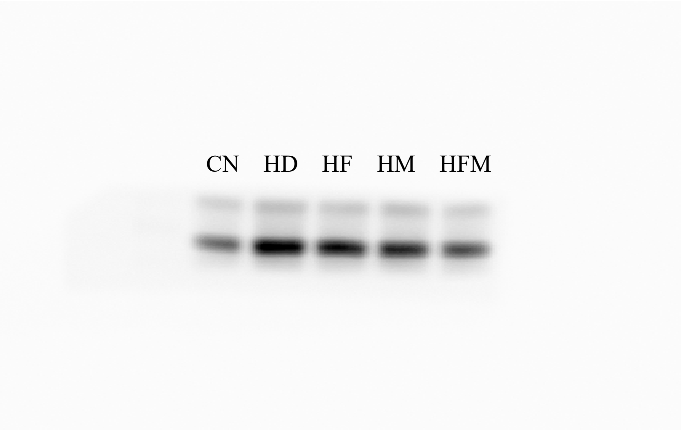

Figure S7. p-eIf-2α in the liver of Western blot. The molecular mass was 38 KD.

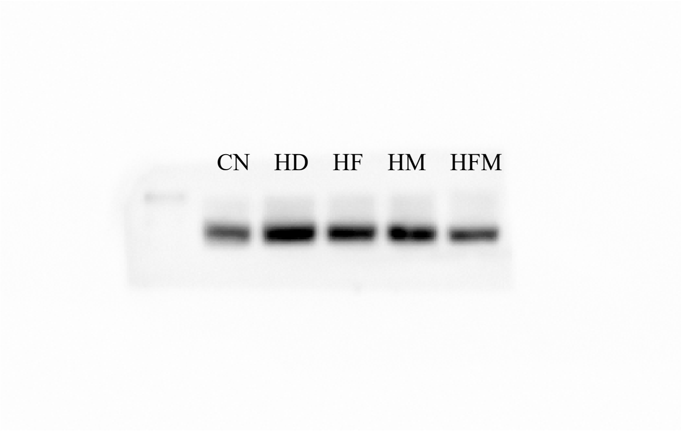

Figure S8. ATF4 in the liver of Western blot. The molecular mass was 38 KD.

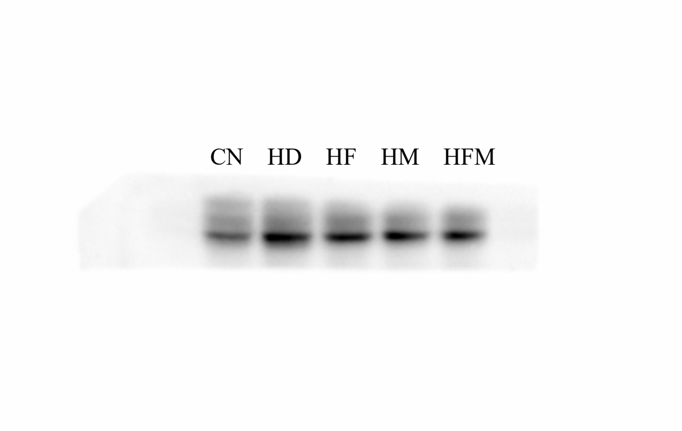

Figure S9. CHOP in the liver of Western blot. The molecular mass was 31 KD.

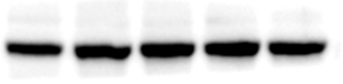

Figure S10. β-actin in the liver of Western blot. The molecular mass was 42 KD.

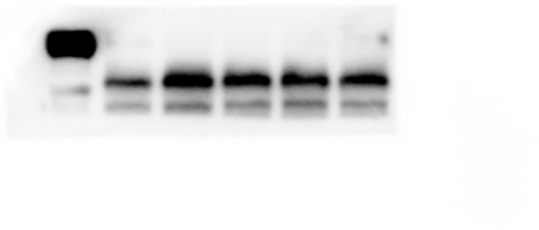

Figure S11. PPARγ in the liver of Western blot. The molecular mass were 57 KD and 53 KD.

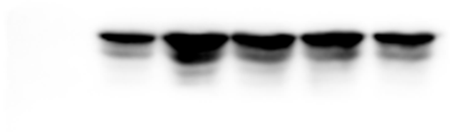

Figure S12. PLIN2 in the liver of Western blot. The molecular mass was 48 KD.

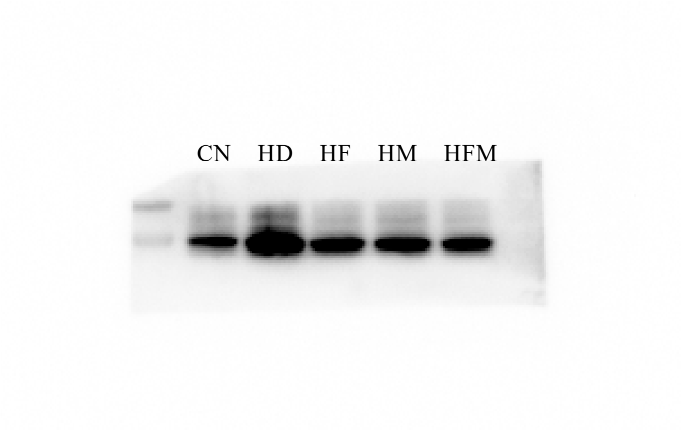

Figure S13. PLIN5 in the liver of Western blot. The molecular mass was 55 KD.

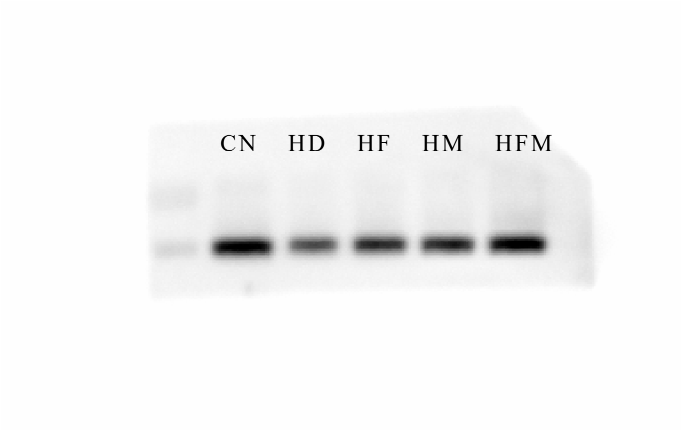

Figure S14. ATGL in the liver of Western blot. The molecular mass was 55 KD.

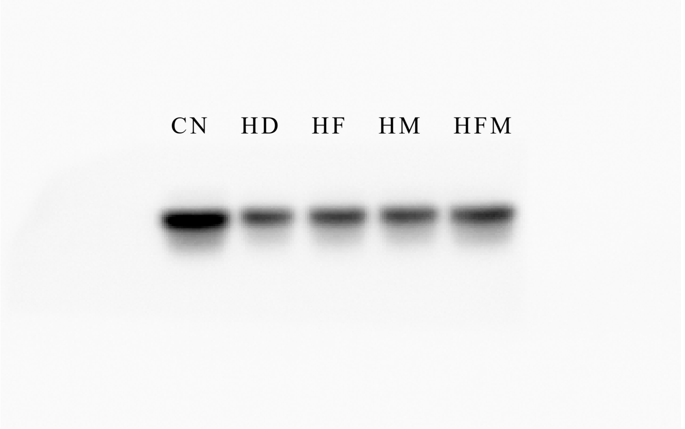

Figure S15. CGI-58 in the liver of Western blot. The molecular mass was 39 KD.

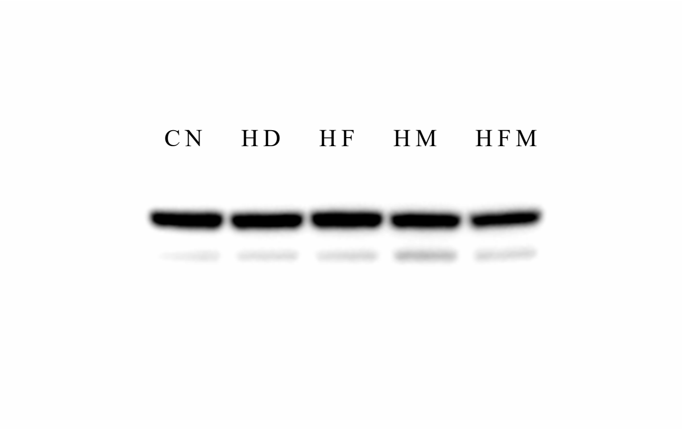

Figure S16. β-actin in the WAT of Western blot. The molecular mass was 42 KD.
